# Reducing Inequalities in Timing of Antenatal Care Initiation: A Hypothetical Intervention in the Generation R Study

**DOI:** 10.1111/ppe.70020

**Published:** 2025-04-06

**Authors:** Clair A. Enthoven, Jeremy A. Labrecque, Hanan El Marroun, Nicole Lucassen, Dionne V. Gootjes, Eefje van Vliet, Hilmar H. Bijma, Pauline W. Jansen

**Affiliations:** ^1^ Department of Child and Adolescent Psychiatry/Psychology Erasmus University Medical Center Rotterdam the Netherlands; ^2^ Department of Psychology, Education and Child Studies Erasmus School of Social and Behavioural Sciences, Erasmus University Rotterdam the Netherlands; ^3^ The Generation R Study Group, Erasmus University Medical Center Rotterdam the Netherlands; ^4^ Department of Epidemiology Erasmus University Medical Center Rotterdam the Netherlands; ^5^ Department of Obstetrics and Gynaecology, Division of Obstetrics and Fetal Medicine Erasmus MC Sophia Rotterdam the Netherlands; ^6^ Department of Care Ethics University of Humanistic Studies Utrecht the Netherlands

**Keywords:** antenatal care, family planning, hypothetical intervention, inequalities, pregnancy recognition

## Abstract

**Background:**

Much research has focused on identifying predictors of late antenatal care initiation. Many of these predictors (e.g., young age, migration background, socioeconomic position) are impossible to modify, illustrating the need to explore other interventions.

**Objectives:**

This study aims to investigate inequalities in antenatal care initiation and assess whether early pregnancy recognition may reduce these inequalities.

**Methods:**

Data from Generation R were used (*N* = 4196), a population‐based birth cohort study in Rotterdam, The Netherlands. The association of gestational age at pregnancy recognition with the timing of antenatal care initiation and associations of individual and socioeconomic factors with the timing of antenatal care initiation were assessed using linear regression analyses. G‐methods were used to estimate the reduction of the inequalities in antenatal care initiation if everyone would have recognised the pregnancy within 6 weeks.

**Results:**

Participants who recognised their pregnancy within 6 weeks (81.7%) had their first antenatal care visit 1.3 weeks (95% confidence interval [CI] −1.6, −0.9) earlier than those who recognised their pregnancy after 6 weeks. All individual and socioeconomic factors were associated with the timing of antenatal care initiation. Modelling a scenario where pregnancy recognition occurred within 6 weeks reduced inequalities in antenatal care initiation timing across several groups: age < 20 versus 30–35 (−0.4 weeks, 95% CI −0.7, −0.2), first‐generation migrants versus no migration (−0.1 weeks, 95% CI −0.2, 0.0), unplanned/ambivalent versus planned pregnancies (−0.4 weeks, 95% CI −0.6, −0.2), lower versus higher educational attainment (−0.1 weeks, 95% CI −0.2, 0.0), unemployed versus employed (−0.1 weeks, 95% CI −0.2, 0.0), low versus high household income (−0.1 weeks, 95% CI −0.3, 0.0), renters versus homeowners (−0.1 weeks, 95% CI −0.2, 0.0), and high versus low neighbourhood deprivation (−0.1 weeks, 95% CI −0.2, −0.1).

**Conclusions:**

Early recognition of pregnancy may help reduce the impact of socio‐economic inequalities in the timely initiation of antenatal care.

## 1 Background

For long, it is known that early and adequate prenatal care is essential to reduce perinatal complications, such as preterm birth [1, 2]. The World Health Organization recommends at least eight visits, and the first visit should be within thirteen weeks of pregnancy [3]. Early antenatal care is important to assess the gestational age, which improves the management of preterm birth, prolonged pregnancy or complications later in pregnancy [4]. Early care initiation is also essential for timely screening of anaemia and rubella, assessing Rh and AB0 blood typing, offering non‐invasive genetic testing and addressing potential risk factors such as high body mass index and domestic violence [5]. Furthermore, considering the tremendous impact of circumstances during pregnancy, timely education of pregnant people regarding healthy behaviours, including nutrition, supplement intake, smoking and alcohol cessation and exercise, is similarly essential [5].

In the Netherlands, antenatal care is generally accessible. All expenses, except travel costs, are covered by the basic health care fund, and specific government arrangements guarantee free obstetric care for pregnant people without health insurance [6]. Still, in 40% of the births in Rotterdam, a multi‐ethnic city with considerable social and perinatal health inequalities [7], antenatal care started only after ten weeks of pregnancy [8]. Previous research pointed at several predictors for a late antenatal care initiation, including young age, single parenthood, lower socio‐economic position, migration background, mental health problems, poorer language ability and higher parity [9, 10, 11, 12, 13, 14]. Notably, many of these factors are also associated with an increased risk of preterm birth [15, 16]. These findings underline the ‘Inverse Care Law’, describing that those most in need of care often have the least access to it [17].

Thus far, most studies have evaluated a wide range of predictors for late antenatal care initiation without discerning between factors that are and are not modifiable [9, 10, 11, 12, 13, 14]. A potentially important modifiable reason for late antenatal care initiation is late recognition of the pregnancy [18, 19]. A study in the United States showed that participants who recognised the pregnancy early (within 6 weeks after the first day of the last menstrual period) were 6 times more likely to initiate antenatal care timely (< 12 weeks of gestation) than those with later pregnancy recognition, after adjustment for socioeconomic variables [18]. Therefore, this study aims to investigate predictors in the timing of antenatal care initiation and assess whether some of these inequalities would be reduced had everyone recognised the pregnancy early.

## 2 Methods

### 2.1 Study Population

The Generation R Study is a multi‐ethnic, population‐based, prospective cohort from foetal life onwards. This cohort has previously been described in detail [20, 21]. Briefly, all pregnant women residing in Rotterdam with a planned delivery date between April 2002 and January 2006 were invited to participate, and 9778 (response rate 61%) women enrolled. All participants with information on gestational age at antenatal care initiation were included, leading to an analytical sample of 4196 participants (Figure S1). Most study variables were obtained via questionnaires available in Dutch, English, and Turkish. Additional support for verbal translation was available in Arabic, Portuguese, and French, if needed [22].

### 2.2 Measures

#### 2.2.1 Antenatal Care Initiation

Information about the timing of antenatal care initiation was derived from electronic patient files (Micronatal) of 23 midwifery practices in Rotterdam for participants with an expected date of birth between April 2002 and December 2004 [23, 24]. The gestational age was determined by ultrasound. The gestational age at antenatal care initiation was used as a continuous variable indicating the gestational age in weeks at the first visit. We additionally created a dichotomous variable indicating whether initiation was within or after 14 weeks' gestation, which illustrates the number of pregnancies that did not meet the recommendations of the Dutch Society of Obstetrics and Gynaecology (NVOG) for basic antenatal care at the time of data collection [25].

#### 2.2.2 Time of Pregnancy Recognition

Gestational age at pregnancy recognition was measured using a self‐report questionnaire at the first research visit. Participants reported to the question: ‘At how many weeks of pregnancy did you know you were pregnant? (based on the first day of your last menstrual period or pregnancy dating)’. Early pregnancy recognition was defined as recognition within 6 weeks since the first day of the last menstrual period.

#### 2.2.3 Predictors

Individual predictors included age, relationship status, migration background, pregnancy intention, mental illness, Dutch language skills and parity since they were previously identified as predictors for late antenatal care initiation [9, 10, 11, 12, 13]. Other variables are socioeconomic factors, such as education, employment, household income, housing and neighbourhood deprivation [14]. In addition, we studied cognitive functioning because it is related to someone's health literacy skills [26]. A detailed description of the measured predictors can be found in Table S1.

### 2.3 Statistical Analyses

The inequalities, that is the associations between the predictors and timing of antenatal care initiation, were estimated using linear regression models. The reference level was the predictor level with the earliest antenatal care initiation, for example ‘30–35 years’ in age, or ‘high’ in educational attainment, to ensure that the inequality was always positive to simplify the interpretation. We were not interested in a potential causal effect of the predictors on timing of antenatal care initiation via a mechanism of timing of pregnancy recognition, which would be obtained when using mediation methods. From a public health point of view, it would be too complicated or even impossible to intervene on age, relationship status, migration background or socioeconomic position for people to initiate antenatal care earlier. Early pregnancy recognition might be a factor that may have a causal relationship with timing of antenatal care initiation, is amenable to intervention and may reduce inequalities in antenatal care initiation. We therefore assessed the effect of early pregnancy recognition on timing of antenatal care initiation using linear regression analyses adjusted for all predictors (age, migration background, relationship status, pregnancy intention, mental illness, Dutch language skills, parity, education, employment, household income, housing, neighbourhood deprivation and cognitive functioning). We used this model to estimate the reduction in inequalities in antenatal care initiation if everyone in our study population had recognised the pregnancy early (i.e., within 6 weeks). By comparing the timing of antenatal care initiation with the hypothetical intervention (i.e., had all participants recognised the pregnancy within 6 weeks) and without the hypothetical intervention (i.e., timing of pregnancy recognition as it appears in the data), the reduction in the inequalities driven by the predictors was estimated [27]. Bootstrapping with 1000 iterations was used to calculate the 95% confidence intervals (CI). This design is also called a hypothetical intervention [27] because we used data from an observational study to estimate the counterfactuals, that is what would have happened if we had intervened so that all participants would recognise the pregnancy within 6 weeks of gestation.

Three causal assumptions apply with respect to the intervention and the outcome [28]. First, exchangeability refers to no residual confounding or bias in the association between pregnancy recognition and the timing of antenatal care initiation. We, therefore, adjusted for all predictors as potential confounders. Second, positivity means that the probability of receiving the intervention (early pregnancy recognition) conditional on the covariates must be greater than 0. This was tested by estimating propensity scores in a model of the non‐imputed dataset with early pregnancy recognition as a dichotomous outcome and all the covariates as predictors. As the participant with the smallest propensity score had a score of 0.217 to receive the intervention, the positivity assumption was satisfied. Third, consistency requires the intervention to be clearly defined. In practice, our intervention would require sexually active participants of reproductive age to take a pregnancy test in case of pregnancy suspicion (e.g., their menstruation did not start in the week that they expected). This would be complicated for those with an irregular cycle, thereby potentially violating the consistency assumption. To relax this assumption, we repeated the analyses in only those with a regular cycle (28 days plus or minus 4 days) in sensitivity analyses [29]. All analyses were conducted in IBM SPSS version 28 and R statistical software version 4.2.1.

### 2.4 Missing Data

Before analyses, multiple imputations were performed to replace the missing values in the predictors (ranging from 1.1% to 36.2%) and timing of pregnancy recognition (25.5%) using multivariate imputation by chained equations [30]. We created 50 imputed datasets with 100 iterations. In addition to the study variables, we used body mass index at intake, gestational age at intake, and Apgar score 5 min after birth as predictors for imputation [31].

### 2.5 Ethics Approval

The Medical Ethics Committee of Erasmus MC in Rotterdam, the Netherlands, has approved the study following the Declaration of Helsinki of the World Medical Association (December 17, 2001, MEC 198.782/2001/31). Written informed consent was obtained from all participants.

## 3 Results

The participants were, on average, 29.1 (SD 5.3) years old. The first antenatal care visit was, on average, at 12.9 (SD 3.7) weeks, and 81.4% of the participants started antenatal care in the first 14 weeks of pregnancy. The pregnancy was, on average, recognised at 5.4 (SD 2.3) weeks, and most participants recognised their pregnancy within 6 weeks. A detailed description of the analytical sample and the total study population is shown in Table 1 and Table S2.

**TABLE 1:** General characteristics of the participants (non‐imputed dataset).

	Total (*N* = 4196)	Antenatal care visit ≤ 14 weeks (*n* = 3417)	Antenatal care visit > 14 weeks (*n* = 779)
	Number (%)	Number (%)	Number (%)
Individual predictors			
Age (years)			
< 20	239 (5.8)	140 (4.2)	99 (12.8)
20–25	769 (18.5)	584 (17.3)	185 (23.8)
25–30	1142 (27.5)	940 (27.9)	202 (26.0)
30–35	1506 (36.3)	1319 (39.1)	187 (24.1)
≥ 35	490 (11.8)	387 (11.5)	103 (13.3)
Migration background			
No	1908 (49.4)	1711 (53.2)	197 (30.3)
2nd generation	610 (15.8)	498 (15.5)	112 (17.2)
1st generation	1348 (34.9)	1007 (31.3)	341 (52.5)
Relationship status			
With partner	3196 (84.2)	2725 (86.4)	471 (73.7)
Single	598 (15.8)	430 (13.6)	168 (26.3)
Pregnancy Intention			
Planned	2454 (69.0)	2139 (72.4)	315 (52.2)
Unplanned and wanted	638 (17.9)	491 (16.6)	147 (24.4)
Unplanned and ambivalent	465 (13.1)	324 (11.0)	141 (23.4)
Mental illness			
No	1845 (61.9)	1559 (61.4)	286 (65.0)
Ever	770 (25.8)	673 (26.5)	97 (22.0)
Recent	365 (12.2)	308 (12.1)	57 (13.0)
Dutch language skills			
Sufficient	2400 (68.2)	2085 (71.0)	315 (54.1)
Reasonable	728 (20.7)	578 (19.7)	150 (25.8)
Insufficient	391 (11.1)	274 (9.3)	117 (20.1)
Socioeconomic predictors			
Educational attainment			
High	910 (24.1)	815 (25.9)	95 (15.0)
Medium	1892 (50.1)	1605 (51.0)	287 (45.3)
Low	977 (25.9)	726 (23.1)	251 (39.7)
Employment			
Yes	2174 (71.6)	1935 (75.1)	239 (52.0)
No	862 (28.4)	641 (24.9)	221 (48.0)
Household income			
High	1886 (59.5)	1704 (63.4)	182 (37.6)
Medium	598 (18.9)	490 (18.2)	108 (22.3)
Low	686 (21.6)	492 (18.3)	194 (40.1)
Housing			
Own home	1626 (50.8)	1466 (54.0)	160 (33.1)
Rented home	1572 (49.2)	1249 (46.0)	323 (66.9)
Neighbourhood deprivation			
Low	1336 (32.2)	1181 (35.0)	155 (20.0)
Medium	1367 (33.0)	1134 (33.6)	233 (30.1)
High	1445 (34.8)	1058 (31.4)	387 (49.9)
Cognitive functioning (IQ)			
≥ 85	2001 (74.8)	1759 (77.4)	242 (60.3)
70–85	521 (19.5)	405 (17.8)	116 (28.9)
< 70	153 (5.7)	110 (4.8)	43 (10.7)
Pregnancy awareness			
Early pregnancy recognition^a^			
No	572 (18.3)	410 (15.8)	162 (30.2)
Yes	2554 (81.7)	2180 (84.2)	374 (69.8)
Menstrual cycle			
Irregular or unknown	1836 (43.8)	1408 (41.2)	428 (54.9)
Regular	2360 (56.2)	2009 (58.8)	351 (45.1)

^a^ Early pregnancy recognition was defined as recognition within 6 weeks since the first day of the last menstrual period.

Participants who recognised their pregnancy within 6 weeks had their first visit in antenatal care 1.3 weeks earlier (95% CI −1.6, −0.9) than those who recognised their pregnancy later than 6 weeks (β_adjusted_ − 0.6, 95% CI −1.0, −0.3). In addition, all predictors were associated with the timing of antenatal care initiation (Table 2). The associations were most prominent for age, migration background, pregnancy intention, Dutch language skills, parity, educational attainment, employment, household income and neighbourhood deprivation.

**TABLE 2:** Differences in timing of entry in antenatal care in weeks between the levels of predictors, and the reduction of these differences after implementing a hypothetical intervention on early pregnancy recognition (*N* = 4196).

Predictor	Without intervention	With intervention	Reduction
	Difference β (95% CI)	Difference β (95% CI)	Difference β (95% CI)
Age (years)			
< 20	3.0 (2.3, 3.6)	2.6 (1.9, 3.2)	−0.4 (−0.7, −0.2)
20–25	1.0 (0.7, 1.3)	0.9 (0.6)	−0.1 (−0.2, 0.0)
25–30	0.3 (0.1, 0.6)	0.2 (−0.1, 0.4)	−0.1 (−0.2, −0.1)
30–35	0.0 (Reference)	0.0 (Reference)	0.0 (Reference)
≥ 35	0.7 (0.4, 1.1)	0.6 (0.3, 1.0)	−0.1 (−0.2, 0.0)
Migration background			
No	0.0 (Reference)	0.0 (Reference)	0.0 (Reference)
2nd generation	1.0 (0.6, 1.3)	0.8 (0.5, 1.2)	−0.1 (−0.2, 0.0)
1st generation	1.5 (1.3, 1.8)	1.4 (1.2, 1.7)	−0.1 (−0.2, 0.0)
Relationship status			
With partner	0.0 (Reference)	0.0 (Reference)	0.0 (Reference)
Single	1.4 (1.1, 1.8)	1.3 (1.0, 1.7)	−0.1 (−0.3, 0.0)
Pregnancy Intention			
Planned	0.0 (Reference)	0.0 (Reference)	0.0 (Reference)
Unplanned and wanted	1.1 (0.8, 1.5)	1.0 (0.7, 1.3)	−0.1 (−0.2, 0.0)
Unplanned and ambivalent	2.1 (1.8, 2.5)	1.8 (1.4, 2.2)	−0.4 (−0.6, −0.2)
Mental illness			
No	0.3 (0.0, 0.7)	0.4 (0.0, 0.7)	0.0 (−0.1, 0.1)
Ever	0.0 (Reference)	0.0 (Reference)	0.0 (Reference)
Recent	0.4 (1.2, 0.6)	0.4 (0.1, 0.6)	0.0 (−0.1, 0.1)
Dutch language skills			
Sufficient	0.0 (Reference)	0.0 (Reference)	0.0 (Reference)
Reasonable	0.7 (0.3, 1.0)	0.7 (0.3, 1.1)	0.0 (−0.1, 0.1)
Insufficient	1.3 (0.8, 1.8)	1.2 (0.7, 1.7)	−0.1 (−0.3, 0.1)
Parity			
0	0.0 (Reference)	0.0 (Reference)	0.0 (Reference)
1	0.1 (−0.1, 0.4)	0.2 (−0.1, 0.4)	0.0 (0.0, 0.1)
2	0.8 (0.4, 1.2)	0.8 (0.4, 1.2)	0.0 (−0.2, 0.1)
≥ 3	1.6 (0.9, 2.3)	1.4 (0.7, 2.2)	−0.2 (0.5, 0.2)
Educational attainment			
High	0.0 (Reference)	0.0 (Reference)	0.0 (Reference)
Medium	0.4 (0.2, 0.7)	0.4 (0.2, 0.7)	0.0 (−0.1, 0.0)
Low	1.5 (1.2, 1.8)	1.4 (1.1, 1.7)	−0.1 (−0.2, 0.0)
Employment			
Yes	0.0 (Reference)	0.0 (Reference)	0.0 (Reference)
No	1.6 (1.4, 1.9)	1.5 (1.2, 1.7)	−0.1 (−0.2, 0.0)
Household income			
High	0.0 (Reference)	0.0 (Reference)	0.0 (Reference)
Medium	0.9 (0.7, 1.2)	0.8 (0.6, 1.1)	−0.1 (−0.2, 0.0)
Low	2.1 (1.8, 2.3)	1.9 (1.6, 2.2)	−0.1 (−0.3, 0.0)
Housing			
Own home	0.0 (Reference)	0.0 (Reference)	0.0 (Reference)
Rented home	1.2 (1.0, 1.5)	1.2 (0.9, 1.4)	−0.1 (−0.2, 0.0)
Neighbourhood deprivation			
Low	0.0 (Reference)	0.0 (Reference)	0.0 (Reference)
Medium	0.7 (0.4, 0.9)	0.6 (0.3, 0.8)	−0.1 (−0.2, 0.0)
High	1.5 (1.2, 1.8)	1.4 (1.1, 1.7)	−0.1 (−0.2, −0.1)
Cognitive functioning (IQ)			
> 85	0.0 (Reference)	0.0 (Reference)	0.0 (Reference)
70–85	0.8 (0.5, 1.1)	0.8 (0.5, 1.1)	0.0 (−0.1, 0.1)
< 70	1.4 (0.9, 1.9)	1.4 (0.9, 1.8)	0.0 (−0.2, 1.1)

**FIGURE 1:**
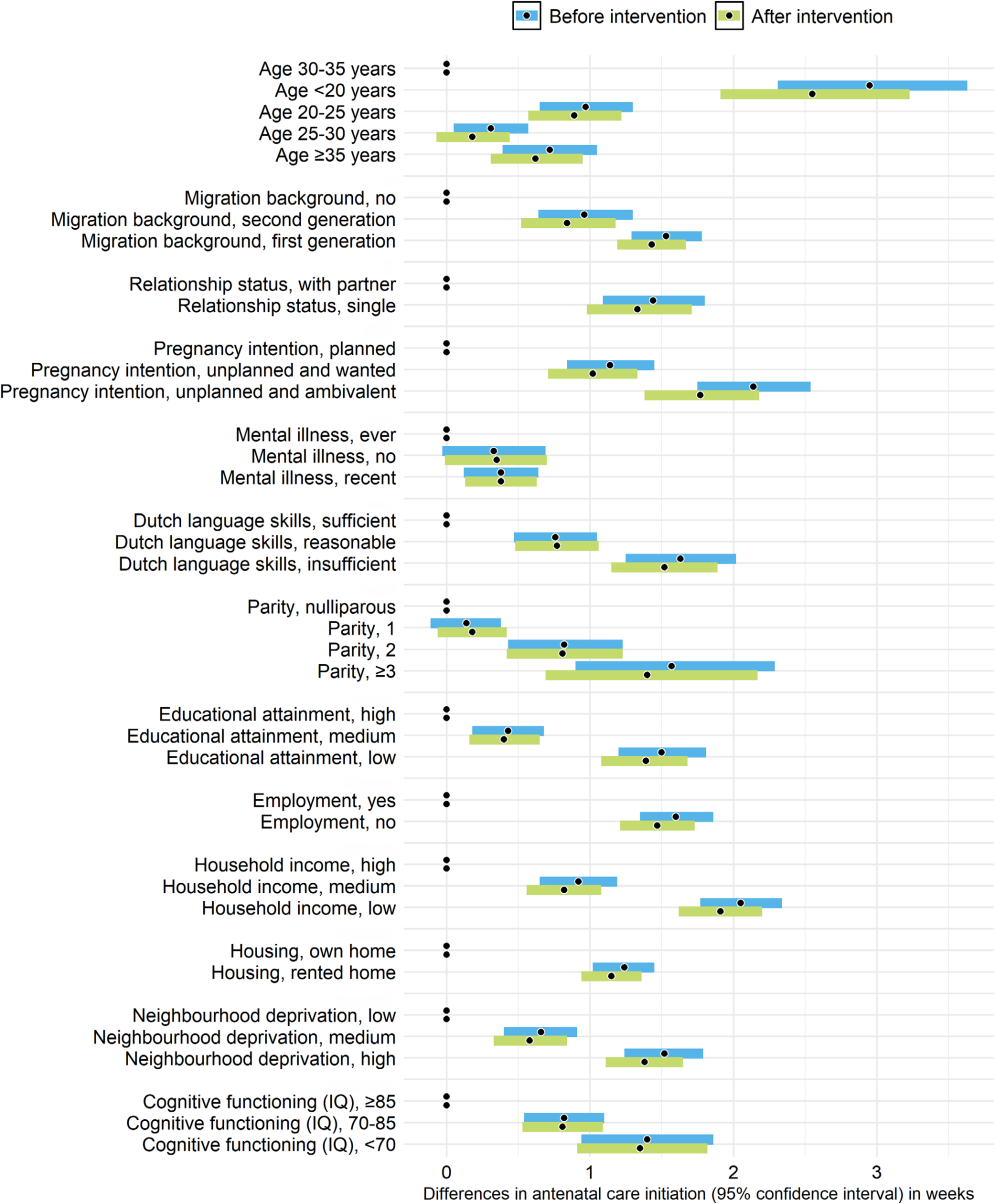
Differences in timing of antenatal care initiation in weeks between the levels of predictors, and the reduction of these differences after implementing a hypothetical intervention on early pregnancy recognition (*N* = 4196). The black dots indicate the beta‐coefficients before (with dark blue confidence intervals) and after (with light blue confidence intervals) implementing the hypothetical intervention. The black dots without confidence intervals (positioned at 0) indicate the reference groups.

Hypothetically, intervening on pregnancy recognition (≤ 6 weeks) reduced the inequalities in antenatal care initiation. The most substantial reductions were observed for age, migration background, pregnancy intention, education, employment, household income, housing and neighbourhood deprivation. Table 2 and Figure 1 show the inequality before and after the intervention and the corresponding reductions.

Sensitivity analyses that include only participants with a regular cycle (*n* = 2360) are shown in Table S3. Participants who recognised their pregnancy within 6 weeks had their first visit in antenatal care 1.1 weeks (95% CI −1.6, −0.7) earlier than those who recognised their pregnancy after 6 weeks (β_adjusted_ = −0.5, 95% CI −0.9, 0.0). All predictors remained associated with the timing of antenatal care initiation. Hypothetically intervening on pregnancy recognition reduced the inequalities for pregnancy intention, migration background and neighbourhood deprivation.

## 4 Comment

### 4.1 Principal Findings

The aims of this study were to investigate predictors for late antenatal care initiation and to assess whether hypothetically intervening in early pregnancy recognition could reduce inequalities in the timing of antenatal care initiation. Our study showed that almost one out of five pregnant participants started antenatal care after 14 weeks of pregnancy during the study period (2002–2006) in Rotterdam. Participants who recognised their pregnancy within the first six weeks had their first visit in antenatal care on average 4.4 days earlier than those who recognised their pregnancy later than 6 weeks. All examined predictors were associated with the timing of antenatal care initiation, with the strongest associations for age, migration background, pregnancy intention, parity, educational level, household income, employment, neighbourhood deprivation and Dutch language skills. Hypothetically, intervening on early pregnancy recognition ≤ 6 weeks may reduce the socioeconomic inequalities.

### 4.2 Strengths of the Study

Strengths of this study include the novel statistical methods, the large availability of relevant variables and the large, representative sample. The analytical sample (*n* = 4196) had characteristics similar to the total population (*N* = 9778; Table S2), eliminating the need for propensity score weighting. Although inclusion in Generation R was aimed at the first trimester, enrolment remained possible until birth, ensuring that those with a late antenatal care initiation were still eligible for participation [20].

### 4.3 Limitations of the Data

The reliance on self‐reported pregnancy recognition limited the analyses. This may be subject to recall bias or socially acceptable answers, which may have diluted the associations. In addition, we lacked information about the participants' knowledge concerning antenatal care and sexual and reproductive health in general, which may be a confounder in the intervention model. Furthermore, one of the assumptions of this causal study design is consistency, which means that the hypothetical intervention must be well‐defined. In practice, an intervention that ensures everyone will recognise the pregnancy within 6 weeks does not exist, potentially violating the consistency assumption. To reduce the possibility of bias, we performed sensitivity analyses among participants who had a regular cycle because they would be more likely to know when they missed their period. The sensitivity analyses showed only reductions in inequalities in antenatal care initiation for pregnancy intention, migration background and neighbourhood deprivation, indicating that our initial results may have been slightly overestimated. Finally, this study was conducted in a country with universal access to healthcare for about 99% of the population [32]. As a result, the findings may be less generalisable to areas without universal healthcare, where barriers such as financial and geographical accessibility may play a larger role [33].

### 4.4 Interpretation

In line with other studies, our results suggest that the inequality in antenatal care initiation could be reduced if the pregnancy was recognised in a timely manner [18, 19]. Most (> 80%) of our participants recognised the pregnancy within 6 weeks, and they initiated antenatal care 4.4 days earlier. Early care is essential to provide prompt education on healthy behaviours and to assess gestational age and embryonal growth in a timely manner, which strongly impacts pregnancy outcomes [4, 5, 34, 35]. Most people use home tests to detect a pregnancy [36]. Home pregnancy tests are readily available in drug stores, pharmacies, grocery stores and online retailers. Still, ensuring timely recognition may be challenging for several reasons. Firstly, some individuals may face barriers to accessing home pregnancy tests due to costs of purchasing them or the ability to reach a store [36]. Secondly, even though most manufacturers claim that home pregnancy tests are 99% reliable, this is lower in practice due to user errors or testing too early [37]. Thirdly, recognising a pregnancy requires knowledge of the reproductive system, which may be more limited in people with lower socioeconomic position [38, 39, 40]. Finally, some people may feel ambivalent about a potential pregnancy and delay testing due to fear of the result or the need for time to consider the options if pregnant [36]. In a recent pilot study, free home pregnancy tests were provided to people with increased risk of delayed pregnancy recognition (i.e., young people, people with irregular menses and those who recently had a second trimester induced abortion). The results showed that self‐testing was feasible and acceptable, with many participants expressing an intention to continue testing after the study [41]. Together, these and our results suggest that providing information and free home pregnancy tests to a specific group of people with an increased risk of delayed pregnancy recognition may help to reduce inequalities in the timing of antenatal care initiation.

The results of our study suggest that early pregnancy recognition may partly reduce inequalities in antenatal care initiation, suggesting that other mechanisms may also occur. Based on analyses in the same dataset as the current study, Choté et al. (2011) focused on ethnic inequalities in early antenatal care initiation. They reported that mothers with a migration background were more likely to be less educated, less likely to be employed, and relatively more likely to be multiparous. These factors were also predictors for a later antenatal care initiation in our study. Additional adjustments for enabling factors (educational level and employment), predisposing factors (age, parity, relationship status, pregnancy intention) and behavioural factors (folic acid intake, maternal smoking, alcohol use) showed a reduction in the ethnic inequalities in antenatal care initiation [25]. Together with our findings, this suggests that antenatal care initiation is affected by a complex interplay of many different factors, causal factors of which are hard to distinguish from correlational factors.

Other explanations underlying inequalities are mainly derived from qualitative studies. The results of these studies suggested that some people postpone their first appointment because they felt healthy, had other responsibilities or did not feel it was urgent [19, 42]. This is in line with research from non‐Western countries showing that some people do not perceive a pregnancy as a health condition [43]. In addition, some people want to avoid antenatal care because they are afraid of judgement or stigma [19]. This occurs notably for young people and those with ambivalent feelings towards an (unintended) pregnancy [19], two predictors that were also identified in our study. Finally, having difficulties navigating antenatal healthcare, particularly when encountering a language barrier as identified in our study, may also lead to delayed access [42]. These mechanisms may inadvertently appear to place the responsibility for late antenatal care initiation on women. However, a Cochrane review showed that one of the most important factors in using antenatal care is the perception of having a positive experience, meaning that care is caring, culturally sensitive, and respectful [44]. The same review also suggested that healthcare providers want to deliver care in such a way, but they are sometimes limited by lack of time (due to organisational tasks) and training. Therefore, improving the healthcare structure, using professional interpreters, increasing the cultural sensitivity of professionals and offering a range of social services may be needed to ensure that the care provided meets the needs of pregnant people [45].

## 5 Conclusions

In this population‐based birth cohort in Rotterdam, significant inequalities in antenatal care initiation were observed, many of which were driven by socioeconomic factors. These disparities are concerning as they may contribute to the persistence or even exacerbation of health inequalities across generations. Importantly, our results suggest that intervening on early pregnancy recognition may reduce, though not eliminate, these inequalities. Therefore, it is recommended that people at risk of unintended pregnancy and people from socioeconomically disadvantaged groups be supported in early pregnancy recognition, for example by providing free tests and education, which is also in line with promoting preconception care.

## Author Contributions

C.A.E., J.A.L., P.W.J. and H.E.M. designed the study. C.A.E. conducted the data analyses and drafted the manuscript. All authors reviewed and revised the study proposal, contributed to data interpretation and critically reviewed and revised the manuscript. All authors approved the final version for publication.

## Conflicts of Interest

The authors declare no conflicts of interest.

## Supporting information


Data S1.

## Data Availability

The data underlying this article cannot be shared publicly because participants of the Generation R study were assured that raw data would remain confidential and would not be shared with the public. However, the data are available on request with a formal data‐sharing agreement for researchers who meet the criteria for access to confidential data. Requests should be directed towards the management team of the Generation R study (secretariaat.genr@erasmusmc.nl). The R script used in this study can be found at https://github.com/centhoven/antenatal_care_initiation.

## References

[1] E. Knight , M. Morris , and M. Heaman , “A Descriptive Study of Women Presenting to an Obstetric Triage Unit With no Prenatal Care,” Journal of Obstetrics and Gynaecology Canada 36, no. 3 (2014): 216–222. 24612890 10.1016/S1701-2163(15)30629-0

[2] E. Papiernik , D. Maine , D. Rush , and A. Richard , “Prenatal Care and the Prevention of Preterm Delivery,” International Journal of Gynecology & Obstetrics 23, no. 5 (1985): 427–433. 2866995 10.1016/0020-7292(85)90153-5

[3] World Health Organization , “WHO Recommendations on Antenatal Care for a Positive Pregnancy Experience: Summary: Highlights and Key Messages From the World Health Organization's 2016 Global Recommendations for Routine Antenatal Care: World Health Organization,” 2018.

[4] J. P. Neilson , P. Cochrane , and G. Childbirth , “Ultrasound for Fetal Assessment in Early Pregnancy,” Cochrane Database of Systematic Reviews 2010, no. 1 (1996): CD000182. 10.1002/14651858.CD000182 10796174

[5] C. Kirkham , S. Harris , and S. Grzybowski , “Evidence‐Based Prenatal Care: Part I. General Prenatal Care and Counseling Issues,” American Family Physician 71, no. 7 (2005): 1307–1316. 15832534

[6] S. Jans , X. Westra , M. Crone , E. van den Akker‐van , M. Marle , and M. Rijnders , “Long‐Term Cost Savings With Centering‐Based Group Antenatal Care,” Midwifery 126 (2023): 103829. 37742587 10.1016/j.midw.2023.103829

[7] J. Poeran , S. Denktas , E. Birnie , G. J. Bonsel , and E. A. P. Steegers , “Urban Perinatal Health Inequalities,” Journal of Maternal‐Fetal & Neonatal Medicine 24, no. 4 (2011): 643–646. 20836740 10.3109/14767058.2010.511341

[8] “Perined. Vroeggeboorte en/of te Laag Geboortegewicht (Big 2),” 2020, https://www.waarstaatjegemeente.nl/jive/.

[9] E. C. Baker and D. Rajasingam , “Using Trust Databases to Identify Predictors of Late Booking for Antenatal Care Within the UK,” Public Health 126, no. 2 (2012): 112–116. 22136699 10.1016/j.puhe.2011.10.007

[10] A. Gadson , E. Akpovi , and P. K. Mehta , “Exploring the Social Determinants of Racial/Ethnic Disparities in Prenatal Care Utilization and Maternal Outcome,” Seminars in Perinatology 41, no. 5 (2017): 308–317. 28625554 10.1053/j.semperi.2017.04.008

[11] S. A. Ali , A. A. Dero , S. A. Ali , and G. B. Ali , “Factors Affecting the Utilization of Antenatal Care Among Pregnant Women: A Literature Review,” Journal of Pregnancy and Neonatal Medicine 2, no. 2 (2018): 41–45.

[12] E. I. de Feijen‐ Jong , D. E. M. C. Jansen , F. Baarveld , C. P. van der Schans , F. G. Schellevis , and S. A. Reijneveld , “Determinants of Late and/or Inadequate Use of Prenatal Healthcare in High‐Income Countries: A Systematic Review,” European Journal of Public Health 22, no. 6 (2012): 904–913. 22109988 10.1093/eurpub/ckr164

[13] H. Kapaya , E. Mercer , F. Boffey , G. Jones , C. Mitchell , and D. Anumba , “Deprivation and Poor Psychosocial Support Are Key Determinants of Late Antenatal Presentation and Poor Fetal Outcomes‐A Combined Retrospective and Prospective Study,” BMC Pregnancy and Childbirth 15, no. 1 (2015): 309. 26608259 10.1186/s12884-015-0753-3 PMC4660789

[14] J. A. Cresswell , G. Yu , B. Hatherall , et al., “Predictors of the Timing of Initiation of Antenatal Care in an Ethnically Diverse Urban Cohort in the UK,” BMC Pregnancy and Childbirth 13, no. 1 (2013): 103. 23642084 10.1186/1471-2393-13-103 PMC3652742

[15] M. E. Wallace , P. Mendola , Z. Chen , B. S. Hwang , and K. L. Grantz , “Preterm Birth in the Context of Increasing Income Inequality,” Maternal and Child Health Journal 20, no. 1 (2016): 164–171. 10.1007/s10995-015-1816-9 PMC6211180 26450504

[16] R. H. Kelly , J. Russo , V. L. Holt , et al., “Psychiatric and Substance Use Disorders as Risk Factors for Low Birth Weight and Preterm Delivery,” Obstetrics and Gynecology 100, no. 2 (2002): 297–304. 12151153 10.1016/s0029-7844(02)02014-8

[17] G. Watt , “The Inverse Care Law Today,” Lancet 360, no. 9328 (2002): 252–254. 12133675 10.1016/S0140-6736(02)09466-7

[18] A. B. Ayoola , M. D. Nettleman , M. Stommel , and R. B. Canady , “Time of Pregnancy Recognition and Prenatal Care Use: A Population‐Based Study in the United States,” Birth 37, no. 1 (2010): 37–43. 20402720 10.1111/j.1523-536X.2009.00376.x

[19] R. Haddrill , G. L. Jones , C. A. Mitchell , and D. O. C. Anumba , “Understanding Delayed Access to Antenatal Care: A Qualitative Interview Study,” BMC Pregnancy and Childbirth 14, no. 1 (2014): 207. 24935100 10.1186/1471-2393-14-207 PMC4072485

[20] V. W. V. Jaddoe , J. P. Mackenbach , H. A. Moll , et al., “The Generation R Study: Design and Cohort Profile,” European Journal of Epidemiology 21, no. 6 (2006): 475–484. 16826450 10.1007/s10654-006-9022-0

[21] M. N. Kooijman , C. J. Kruithof , C. M. van Duijn , et al., “The Generation R Study: Design and Cohort Update 2017,” European Journal of Epidemiology 31, no. 12 (2016): 1243–1264. 28070760 10.1007/s10654-016-0224-9 PMC5233749

[22] V. W. V. Jaddoe , C. M. van Duijn , A. J. van der Heijden , et al., “The Generation R Study: Design and Cohort Update 2010,” European Journal of Epidemiology 25 (2010): 823–841. 20967563 10.1007/s10654-010-9516-7 PMC2991548

[23] A. A. Choté , C. J. De Groot , M. A. Bruijnzeels , et al., “Ethnic Differences in Antenatal Care Use in a Large Multi‐Ethnic Urban Population in The Netherlands,” Midwifery 27, no. 1 (2011): 36–41, 10.1016/j.midw.2009.07.008. 19939527

[24] A. Choté , “Ethnic Differences in Antenatal Care Use, Quality of Care and Pregnancy Outcomes: The Generation R Study,” 2011.

[25] A. A. Choté , G. T. Koopmans , W. K. Redekop , et al., “Explaining Ethnic Differences in Late Antenatal Care Entry by Predisposing, Enabling and Need Factors in The Netherlands. The Generation R Study,” Maternal and Child Health Journal 15, no. 6 (2011): 689–699, 10.1007/s10995-010-0619-2. 20533083 PMC3131512

[26] A. D. Federman , M. Sano , M. S. Wolf , A. L. Siu , and E. A. Halm , “Health Literacy and Cognitive Performance in Older Adults,” Journal of the American Geriatrics Society 57, no. 8 (2009): 1475–1480. 19515101 10.1111/j.1532-5415.2009.02347.x PMC2754116

[27] M. Lara , J. A. Labrecque , F. J. van Lenthe , and T. Voortman , “Estimating Reductions in Ethnic Inequalities in Child Adiposity From Hypothetical Diet, Screen Time, and Sports Participation Interventions,” Epidemiology 31, no. 5 (2020): 736–744. 32618712 10.1097/EDE.0000000000001221

[28] M. A. Hernán and J. M. Robins , Causal Inference: What if (Chapman & Hall/CRC, 2020).

[29] D. O. Mook‐Kanamori , E. A. P. Steegers , P. H. Eilers , H. Raat , A. Hofman , and V. W. V. Jaddoe , “Risk Factors and Outcomes Associated With First‐Trimester Fetal Growth Restriction,” Journal of the American Medical Association 303, no. 6 (2010): 527–534. 20145229 10.1001/jama.2010.78

[30] S. Buuren and K. Groothuis‐Oudshoorn , “Mice: Multivariate Imputation by Chained Equations in R,” Journal of Statistical Software 45 (2010): 1–68.

[31] P. C. Austin , I. R. White , D. S. Lee , and S. van Buuren , “Missing Data in Clinical Research: A Tutorial on Multiple Imputation,” Canadian Journal of Cardiology 37, no. 9 (2021): 1322–1331. 33276049 10.1016/j.cjca.2020.11.010 PMC8499698

[32] T. Kuipers , R. van de Pas , and A. Krumeich , “Is the Healthcare Provision in The Netherlands Compliant With Universal Health Coverage Based on the Right to Health? A Narrative Literature Review,” Globalization and Health 18, no. 1 (2022): 38. 35366916 10.1186/s12992-022-00831-7 PMC8976435

[33] D. H. Peters , A. Garg , G. Bloom , D. G. Walker , W. R. Brieger , and R. M. Hafizur , “Poverty and Access to Health Care in Developing Countries,” Annals of the New York Academy of Sciences 1136, no. 1 (2008): 161–171. 17954679 10.1196/annals.1425.011

[34] E. M. van Uitert , N. Exalto , G. J. Burton , et al., “Human Embryonic Growth Trajectories and Associations With Fetal Growth and Birthweight,” Human Reproduction 28, no. 7 (2013): 1753–1761, 10.1093/humrep/det115. 23569080

[35] J. Stephenson , N. Heslehurst , J. Hall , et al., “Before the Beginning: Nutrition and Lifestyle in the Preconception Period and Its Importance for Future Health,” Lancet 391, no. 10132 (2018): 1830–1841. 29673873 10.1016/S0140-6736(18)30311-8 PMC6075697

[36] L. J. Ralph , D. G. Foster , R. Barar , and C. H. Rocca , “Home Pregnancy Test Use and Timing of Pregnancy Confirmation Among People Seeking Health Care,” Contraception 107 (2022): 10–16. 34748750 10.1016/j.contraception.2021.10.006

[37] C. Gnoth and S. Johnson , “Strips of Hope: Accuracy of Home Pregnancy Tests and New Developments,” Geburtshilfe und Frauenheilkunde 74, no. 7 (2014): 661–669. 25100881 10.1055/s-0034-1368589 PMC4119102

[38] A. B. Ayoola , G. L. Zandee , and Y. J. Adams , “Women's Knowledge of Ovulation, the Menstrual Cycle, and Its Associated Reproductive Changes,” Birth 43, no. 3 (2016): 255–262, 10.1111/birt.12237. 27157718

[39] L. S. Lundsberg , L. Pal , A. M. Gariepy , X. Xu , M. C. Chu , and J. L. Illuzzi , “Knowledge, Attitudes, and Practices Regarding Conception and Fertility: A Population‐Based Survey Among Reproductive‐Age United States Women,” Fertility and Sterility 101, no. 3 (2014): 767–774. 24484995 10.1016/j.fertnstert.2013.12.006

[40] W.‐Y. Ip , M.‐Y. Chan , D. S. K. Chan , and C. W. H. Chan , “Knowledge of and Attitude to Contraception Among Migrant Woman Workers in Mainland China,” Journal of Clinical Nursing 20, no. 11–12 (2011): 1685–1695. 21255168 10.1111/j.1365-2702.2010.03404.x

[41] N. Morris , K. Ehrenreich , T. Gurazada , and D. Grossman , “Feasibility and Acceptability of At‐Home Routine Pregnancy Testing in the United States: A Pilot Study,” Women's Health Issues 33, no. 3 (2023): 258–265. 36822914 10.1016/j.whi.2023.01.002

[42] B. Hatherall , J. Morris , F. Jamal , et al., “Timing of the Initiation of Antenatal Care: An Exploratory Qualitative Study of Women and Service Providers in East London,” Midwifery 36 (2016): 1–7. 27106937 10.1016/j.midw.2016.02.017 PMC4853798

[43] D. Warri and A. George , “Perceptions of Pregnant Women of Reasons for Late Initiation of Antenatal Care: A Qualitative Interview Study,” BMC Pregnancy and Childbirth 20, no. 1 (2020): 70. 32013894 10.1186/s12884-020-2746-0 PMC6998826

[44] S. Downe , K. Finlayson , Ö. Tunçalp , and A. M. Gülmezoglu , “Provision and Uptake of Routine Antenatal Services: A Qualitative Evidence Synthesis,” Cochrane Database of Systematic Reviews 6, no. 6 (2019): CD012392. 31194903 10.1002/14651858.CD012392.pub2 PMC6564082

[45] S. Bains , S. Skråning , J. Sundby , S. Vangen , I. K. Sørbye , and B. V. Lindskog , “Challenges and Barriers to Optimal Maternity Care for Recently Migrated Women—A Mixed‐Method Study in Norway,” BMC Pregnancy and Childbirth 21, no. 1 (2021): 686. 34620114 10.1186/s12884-021-04131-7 PMC8495671

