# Supplementary material for: Reducing Inequalities in Timing of Antenatal Care Initiation: A Hypothetical Intervention in the Generation R Study
Source: Paediatr Perinat Epidemiol. 2025 Apr 6;39(6):502–11. doi: 10.1111/ppe.70020 (PMC12391855; doi:10.1111/ppe.70020)
Supplement: Supplementary file 1 — Data S1. [file PPE-39-502-s001.docx]

**Reducing Inequalities in Timing of Antenatal Care Initiation: A Hypothetical Intervention in the Generation R Study**

**Supplemental material**

**
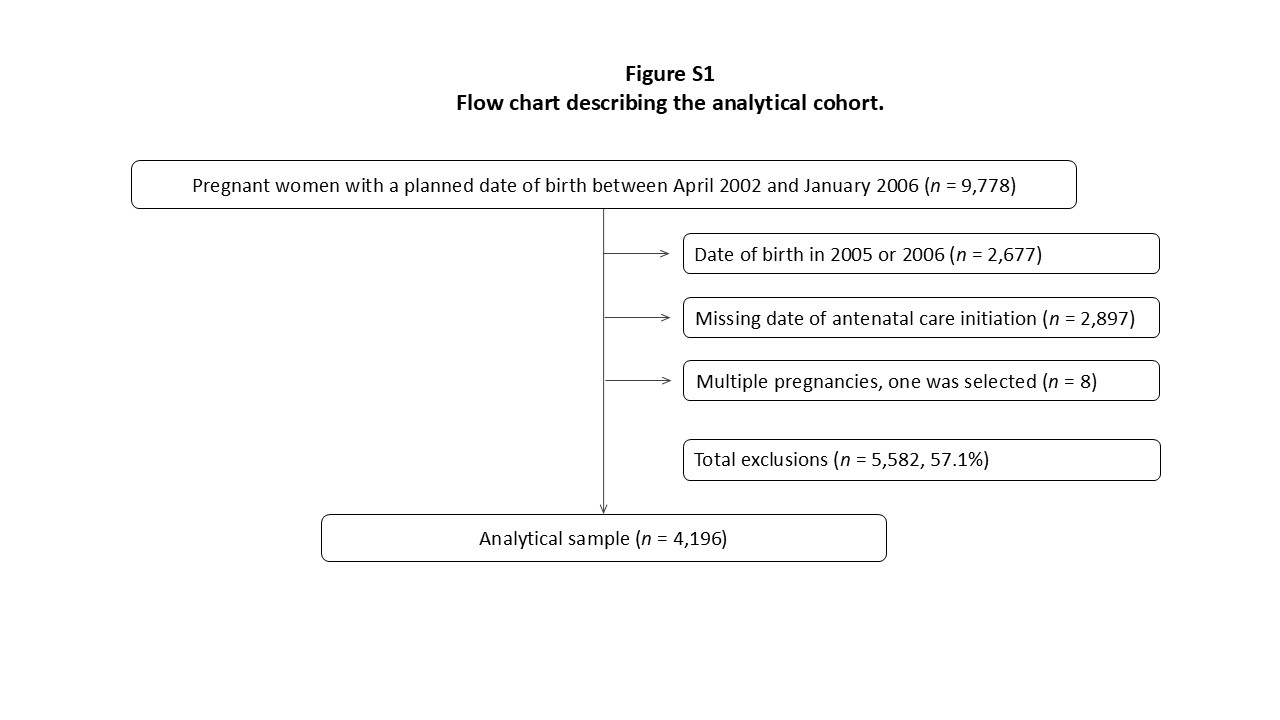
**

**Table S1: Description of the individual and socioeconomic predictors.**

| **Individual predictors** | The individual predictors were measured by questionnaire in the 12^th^ week of gestation, or during enrolment in case participation started after the 12^th^ week of gestation. |
| --- | --- |
| Age at conception | Age at conception was calculated as age at birth of the child minus gestational age at birth and categorized into <20 years, 20-25 years, 25-30 years, 30-35 years and ≥35 years. |
| Migration background | Migration background was defined as one or both parents being born in another country than the Netherlands, according to the classification of Statistics Netherlands.^1^ This was categorized into first- or second-generation migrant depending on whether or not the woman herself was born in the Netherlands.^2, 3^ |
| Relationship status | Relationship status was categorized as being married or cohabiting, versus single. |
| Pregnancy intention | Pregnancy planning was measured using self-report by asking whether the pregnancy was planned or not. In the case of an unplanned pregnancy, women reported how they felt about the pregnancy using the following four answering categories: “pleased from the start”; “initially mixed feelings”; “still mixed feelings”; or “mostly unhappy about the pregnancy”. Pregnancy intention was categorized into planned, unplanned and wanted from the start and unplanned with ambivalent feelings. |
| Mental illness | History and current mental illness were assessed at 20 weeks’ gestation using vignettes explaining depression, anxiety, psychosis, eating disorders and addiction to substances, followed by questions about whether the women had ever experienced these symptoms and received treatment for them, and whether these symptoms were present in the past year. Mental illness was defined as either depression, anxiety, psychosis, eating disorders or substance use addiction and categorised into recent, when the symptoms were present in the last year, or ever, when the symptoms occurred in their lifetime, but not in the last year. |
| Dutch language skills | Proficiency in Dutch language skills was measured by a questionnaire in week 30 of gestation. The pregnant women were asked to rate their Dutch speaking, reading, and writing skills on three separate 5-point scales ranging from ‘not at all’ (1) to ‘good’ (5). This information was summed into general ‘Dutch language skills’ (1–9, not good; 10–14, reasonable; 15, good).^4^ |
| **Socioeconomic predictors** |  |
| Educational attainment | Educational attainment was measured by questionnaire in the 12^th^ week of gestation and defined as the highest attained educational level, categorized as: Low (primary school; lower vocational training; intermediate general school; 3 years general secondary school), which typically corresponds to ≤12 years of education; Medium (>3 years general secondary school; intermediate vocational training; higher vocational training; Bachelor's degree), in general corresponding with 13–17 years of education; and High (higher academic education; PhD), usually indicating 18 years of education or more. |
| Employment | Employment was measured by questionnaire in the 12^th^ week of gestation and categorized into having a paid job (including being self-employed) versus having no paid job. |
| Net household income | Net household income was measured by questionnaire in the 12^th^ week of gestation and categorized as: Less than €1200/month (social security level at the time of assessment); Between €1200 and 2000/month; and More than €2000/month (modal income or more). |
| Housing | Housing was measured by a questionnaire in the 30^th^ week of gestation and categorized into living in a bought or rented home. |
| Neighbourhood deprivation | Neighbourhood deprivation was measured using area-based status scores provided by the Netherlands Institute for Social Research (in Dutch: Sociaal en Cultureel Planbureau).^5^ The scores were matched on four-digit postcodes and are based on mean household income, proportion of the population with low income, proportion of population with low educational attainment and proportion of population without paid work.^6^ The scores were categorized into tertiles (low, medium and high deprivation). |
| Cognitive functioning | Cognitive functioning of the participants was measured using the 12-item validated short version of the Raven's Progressive Matrices at the research centre when their child was 5 years old.^7^ This was categorized into <70 (mild intellectual disability), 70-85 (borderline intellectual functioning) and ≥85 (normal intellectual functioning).^8^ We expect that this measure is relatively stable, and not influenced by the timing of antenatal care initiation. |

**Table S2: General characteristics of the participants in the analytical sample and the full cohort (non-imputed datasets).**

|  | **Analytical sample (N=4,196)** | **Full cohort (N=9,778)** |
| --- | --- | --- |
| **Individual predictors** |  |  |
| **Age (years)** |  |  |
| <20 years | 239 (5.8%) | 467 (4.9%) |
| 20-25 years | 769 (18.5%) | 1,595 (16.7%) |
| 25-30 years | 1,142 (27.5%) | 2,585 (27.1%) |
| 30-35 | 1,506 (36.3%) | 3,497 (36.7%) |
| ≥35 years | 490 (11.8%) | 1,393 (14.6%) |
| **Migration background** |  |  |
| No | 1,908 (49.4%) | 4,544 (51.4%) |
| 2^nd^ generation | 610 (15.8%) | 1,380 (15.6%) |
| 1^st^ generation | 1,348 (34.9%) | 2,919 (33.0%) |
| **Relationship status** |  |  |
| With partner | 3,196 (84.2%) | 7,326 (85.5%) |
| Single | 598 (15.8%) | 1,238 (14.5%) |
| **Pregnancy Intention** |  |  |
| Planned | 2,454 (69.0%) | 5,828 (72.5%) |
| Unplanned and wanted | 638 (17.9%) | 1,219 (15.2%) |
| Unplanned and ambivalent | 465 (13.1%) | 989 (12.3%) |
| **Mental illness** |  |  |
| No | 1845 (61.9%) | 3,833 (62.1%) |
| Ever | 770 (25.8%) | 1,607 (26.0%) |
| Recent | 365 (12.2%) | 732 (11.9%) |
| **Dutch language skills** |  |  |
| Sufficient | 2,400 (68.2%) | 5,094 (68.4%) |
| Reasonable | 728 (20.7%) | 1,546 (20.8%) |
| Insufficient | 391 (11.1%) | 803 (10.8%) |
| **Socioeconomic predictors** |  |  |
| **Educational attainment** |  |  |
| High | 910 (24.1%) | 2,005 (23.4%) |
| Medium | 1,892 (50.1%) | 4,281 (50.0%) |
| Low | 977 (25.9%) | 2,270 (26.5%) |
| **Employment** |  |  |
| Yes | 2,174 (71.6%) | 4,924 (72.6%) |
| No | 862 (28.4%) | 1,863 (27.4%) |
| **Household income** |  |  |
| High | 1,886 (59.5%) | 4,079 (60.8%) |
| Medium | 598 (18.9%) | 1,245 (18.6%) |
| Low | 686 (21.6%) | 1,387 (20.7%) |
| **Housing** |  |  |
| Own home | 1,626 (50.8%) | 3,599 (54.4%) |
| Rented home | 1,572 (49.2%) | 3,011 (45.6%) |
| **Neighbourhood deprivation** |  |  |
| Low | 1,336 (32.2%) | 3,092 (32.1%) |
| Medium | 1,367 (33.0%) | 3,297 (34.2%) |
| High | 1,445 (34.8%) | 3,240 (33.6%) |
| **Cognitive functioning (IQ)** |  |  |
| ≥85 | 2,001 (74.8%) | 4,697 (74.0%) |
| 70-85 | 521 (19.5%) | 1,301 (20.5%) |
| <70 | 153 (5.7%) | 353 (5.6%) |
| **Pregnancy awareness** |  |  |
| **Early pregnancy recognition*** |  |  |
| No | 572 (18.3%) | 1,249 (18.4%) |
| Yes | 2,554 (81.7%) | 5,528 (81.6%) |
| **Menstrual cycle** |  |  |
| Irregular or unknown | 1,836 (43.8%) | 4,709 (48.2%) |
| Regular | 2,360 (56.2%) | 5,069 (51.8%) |

*Early pregnancy recognition was defined as recognition within 6 weeks since the first day of the last menstrual period

**Table S3: Differences in timing of entry in antenatal care in weeks between the levels of predictors, and the reduction of these differences after implementing a hypothetical intervention on early pregnancy recognition, including only participants with a regular cycle (N=2360).**

|  | **Without intervention** | **With intervention** | **Reduction** |
| --- | --- | --- | --- |
| **Predictor** | **Difference β (95% CI)** | **Difference β (95% CI)** | **Difference β (95% CI)** |
| **Age (years)** |  |  |  |
| <20 | 3.06 (2.19; 4.00) | 2.77 (1.92; 3.66) | -0.30 (-0.67; 0.00) |
| 20-25 | 1.34 (0.89; 1.80) | 1.25 (0.82; 1.70) | -0.09 (-0.21; 0.03) |
| 25-30 | 0.24 (-0.05; 0.54) | 0.19 (-0.09; 0.49) | -0.05 (-0.13; 0.03) |
| 30-35 | 0.0 (Reference) | 0.0 (Reference) | 0.0 (Reference) |
| ≥35 | 0.81 (-0.45; 1.18) | 0.73 (0.38; 1.08) | -0.08 (-0.19; 0.01) |
| **Migration background** |  |  |  |
| No | 0.0 (Reference) | 0.0 (Reference) | 0.0 (Reference) |
| 2^nd^ generation | 0.87 (0.49; 1.26) | 0.77 (0.41; 1.16) | -0.10 (-0.24; 0.04) |
| 1^st^ generation | 1.35 (1.05; 1.65) | 1.26 (0.97; 1.56) | -0.09 (-0.17; -0.01) |
| **Relationship status** |  |  |  |
| With partner | 0.0 (Reference) | 0.0 (Reference) | 0.0 (Reference) |
| Single | 1.56 (1.08; 2.07) | 1.44 (0.96; 1.95) | -0.12 (-0.30; 0.05) |
| **Pregnancy Intention** |  |  |  |
| Planned | 0.0 (Reference) | 0.0 (Reference) | 0.0 (Reference) |
| Unplanned and wanted | 1.11 (0.72; 1.51) | 1.00 (0.62; 1.40) | -0.11 (-0.22; 0.00) |
| Unplanned and ambivalent | 2.21 (1.68; 2.78) | 1.79v(1.27; 2.33) | -0.42 (-0.07; -0.17) |
| **Mental illness** |  |  |  |
| No | 0.60 (0.16; 1.05) | 0.55 (0.12; 0.98) | -0.05 (-0.18; 0.07) |
| Ever | 0.0 (Reference) | 0.0 (Reference) | 0.0 (Reference) |
| Recent | 0.41 (0.11; 0.71) | 0.39 (0.10; 0.68) | -0.03 (-0.10; 0.06) |
| **Dutch language skills** |  |  |  |
| Sufficient | 0.0 (Reference) | 0.0 (Reference) | 0.0 (Reference) |
| Reasonable | 0.65 (0.29; 1.03) | 0.68 (0.32; 1.07) | 0.03 (-0.05; 0.12) |
| Insufficient | 1.28 (0.79; 1.78) | 1.19 (0.73; 1.67) | -0.08 (-0.26; 0.06) |
| **Parity** |  |  |  |
| 0 | 0.0 (Reference) | 0.0 (Reference) | 0.0 (Reference) |
| 1 | 0.22 (-0.07; 0.51) | 0.25 (-0.03; 0.54) | 0.04 (-0.04; 0.11) |
| 2 | 0.47 (-0.01; 0.99) | 0.48 (0.00; 1.03) | 0.01 (-0.16; 0.16) |
| ≥3 | 1.55 (0.65; 2.48) | 1.19 (0.32; 2.09) | -0.36 (-0.90; 0.10) |
| **Educational attainment** |  |  |  |
| High | 0.0 (Reference) | 0.0 (Reference) | 0.0 (Reference) |
| Medium | 0.37 (0.07; 0.66) | 0.37 (0.09; 0.65) | 0.01 (-0.06; 0.09) |
| Low | 1.23 (0.86; 1.60) | 1.21 (0.85; 1.57) | -0.03 (-0.14; 0.09) |
| **Employment** |  |  |  |
| Yes | 0.0 (Reference) | 0.0 (Reference) | 0.0 (Reference) |
| No | 1.57 (1.24; 1.91) | 1.48 (1.15; 1.81) | -0.09 (-0.21; 0.01) |
| **Household income** |  |  |  |
| High | 0.0 (Reference) | 0.0 (Reference) | 0.0 (Reference) |
| Medium | 0.69 (0.37; 1.01) | 0.66 (0.36; 0.97) | -0.03 (-0.12; 0.06) |
| Low | 1.96 (1.59; 2.33) | 1.83 (1.47;2.21) | -0.12 (0.25; 0.00) |
| **Housing** |  |  |  |
| Own home | 0.0 (Reference) | 0.0 (Reference) | 0.0 (Reference) |
| Rented home | 1.09 (0.83; 1.35) | 1.03 (0.77; 1.28) | -0.06 (-0.14; 0.01) |
| **Neighbourhood deprivation** |  |  |  |
| Low | 0.0 (Reference) | 0.0 (Reference) | 0.0 (Reference) |
| Medium | 0.59 (0.30; 0.89) | 0.54 (0.26; 0.83) | -0.05 (-0.12; 0.03) |
| High | 1.38 (1.05; 1.71) | 1.27 (0.94; 1.59) | -0.11 (-0.21; -0.03) |
| **Cognitive functioning (IQ)** |  |  |  |
| >85 | 0.0 (Reference) | 0.0 (Reference) | 0.0 (Reference) |
| 70-85 | 0.84 (0.49; 1.20) | 0.82 (0.47; 1.18) | -0.02 (-0.13; 0.08) |
| <70 | 1.22 (0.66; 1.81) | 1.22 (0.67; 1.81) | 0.01 (-0.15; 0.14) |

**References**

1 Netherlands, S. (2004). "Allochtonen in Nederland 2004." Voorburg/Heerlen.

2 Choté, A. A., et al. (2011). "Explaining ethnic differences in late antenatal care entry by predisposing, enabling and need factors in the Netherlands. The Generation R Study." Maternal and Child Health Journal **15**(6): 689-699.

3 Choté, A. A., et al. (2014). "Differences in timely antenatal care between first and second-generation migrants in the Netherlands." Journal of Immigrant and Minority Health **16**(4): 631-637.

4 Jansen, P. W., et al. (2010). "National origin and behavioural problems of toddlers: the role of family risk factors and maternal immigration characteristics." Journal of abnormal child psychology **38**: 1151-1164.

5 Duijn, V. A. N. (2002). "Sociaal-economische status indicator op postcode niveau." Maandstatistiek van de bevolking **50**: 32-35.

6 Gootjes, D. V., et al. (2021). "Association between neighbourhood deprivation, fetal growth, small-for-gestational age and preterm birth: a population-based prospective cohort study." BMJ Open **11**(11): e049075.

7 Irrgang, M., et al. (2019). "C-28 Raven’s Progressive Matrices: Validation of a Short Form." Archives of Clinical Neuropsychology **34**(6): 1057-1057.

8 Nouwens, P. J. G., et al. (2017). "Identifying classes of persons with mild intellectual disability or borderline intellectual functioning: a latent class analysis." BMC Psychiatry **17**(1): 257.
